# A Stress Sensor Based on Galvanic Skin Response (GSR) Controlled by ZigBee

**DOI:** 10.3390/s120506075

**Published:** 2012-05-10

**Authors:** María Viqueira Villarejo, Begoña García Zapirain, Amaia Méndez Zorrilla

**Affiliations:** DeustoTech-Life Unit, Deusto Institute of Technology, University of Deusto, Avda. de las Universidades, 24, Bilbao 48007, Spain; E-Mails: maria.viqueira@opendeusto.es (M.V.V.); mbgarciazapi@deusto.es (B.G.Z.)

**Keywords:** GSR, skin resistance, stress, ZigBee

## Abstract

Sometimes, one needs to control different emotional situations which can lead the person suffering them to dangerous situations, in both the medium and short term. There are studies which indicate that stress increases the risk of cardiac problems. In this study we have designed and built a stress sensor based on Galvanic Skin Response (GSR), and controlled by ZigBee. In order to check the device's performance, we have used 16 adults (eight women and eight men) who completed different tests requiring a certain degree of effort, such as mathematical operations or breathing deeply. On completion, we appreciated that GSR is able to detect the different states of each user with a success rate of 76.56%. In the future, we plan to create an algorithm which is able to differentiate between each state.

## 1. Introduction

Stress, better explained in [1], is a response to particular events. It is the way our body prepares itself to face a difficult situation with focus, strength and heightened alertness. When we perceive a threat, our nervous system responds by releasing a flood of stress hormones, including adrenaline and cortisol. These hormones rouse the body for emergency action. In some cases it is necessary to collect feedback in order to control this symptom because it can become dangerous in certain situations. Therefore, it is necessary to build a device to detect stress.

For this objective, we have designed a Galvanic Skin Response (GSR) device in order to detect the different conductance of the skin when a person is under stress or when not [2]. It uses just two electrodes which are placed on the fingers and act as if they were the two terminals of one resistance [3,4].

**Figure 1.**
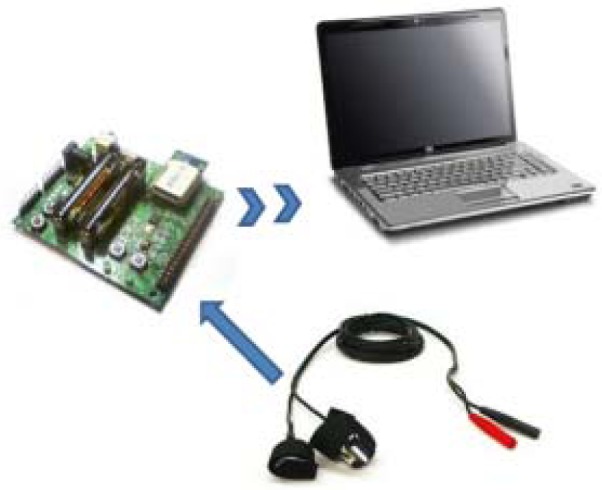
Final application.

This device sends different data to a coordinator via ZigBee and, at the same time, this coordinator will send the information to a computer. The final objective is to implement this GSR into an application which controls different medical devices, [5,6]. The Figure 1 shows the communication of the final application.

The user can use the stress sensor anywhere in his home provided he is at a distance of less than 10 meters [7]. By using a wireless communication system, the user is provided with a certain degree of freedom when using the device. The final user could manage the different devices from his television and the control center could take different action so as to change a person's stress levels. Therefore, the coordination center could use different systems to help the person relax, such as turning down the lights or changing the kind of music the user is listening to. There are two main reasons why we decided to work with ZigBee:

- It's low power consumption.
- It is possible to connect as many as 255 nodes.

This means of communication has been used before in healthcare applications, as can be seen in [8] and [9]. In order to verify the stress sensor, we carried out different trials with 16 adult subjects. The idea was to establish one threshold for each person because there are people who are more nervous than others, so there could be cases where the results are not reliable. Despite this, there exist studies which have obtained good results establishing the same limit for all the subjects [10].

Therefore, there are two parts: the hardware design of the GSR and the algorithm which detects the emotional state of the user. This first study focuses on the hardware part, so the trials were done in order to verify that the device detects different changes in the person's condition, more specifically whether it detects an effort being made.

The output voltage of the designed circuit is connected to the ADC of a ZigBee board. There are two ZigBee boards: one for acquiring the data, and a second one to send it to the computer. This second board (the coordinator) also receives information about other devices and functions of the domotic application.

This paper is divided into the following sections: first, the paper describes the state of the art. Secondly, a complete methodology with all the technologies involved in this system is described, and then the system design is explained, as well as the results obtained during the tests carried out on the system. This document ends with the conclusions and the discussions arising from the topic.

## 2. State of the Art

There exist different studies which try to detect people's emotional states, including attempts to find out whether someone is suffering from stress or is not. Studies [11] and [12] use EEG to classify the different data acquired by brain activity. They extract different frequency features of the signals for their posterior classification with good results.

Heart Rate Variability is another parameter used to measure stress levels [13]. To induce stress the authors in [14] propose using hyperventilation and talk preparation. Then, they present a method based on fuzzy logic to analyze the different data from HR and GSR. An ambulatory device is developed in [4] in order to evaluate stress in blind people. This device also includes the measurement of skin temperature, which is another parameter used to analyze stress [15].

As regards Galvanic Skin Response (GSR), there are several studies which propose different methods of detecting stress levels by measuring skin conductance [16]. The study described in [17] has the objective of detecting sweat levels for the diagnosis of sudomotor dysfunction, something that can help in the diagnosis of diabetes. There are other medical applications based on skin conductance, such as epilepsy control: sweaty hands may be a warning signal of an epileptic attack [18]; or [19], as support of the diagnosis and treatment of bipolar disorder patients.

By combining the sweat of the hands with the temperature of the skin, it is possible to develop a truth meter [20]; as when the person is lying, his hands are colder and skin resistance is lower. In this case it is not necessary to include an ADC because the variation of skin resistance happens at odd times so, with different resistances and transistors, it is possible to build a lie detector.

In [10], different videos are shown to the participants in order to induce different emotions. The data acquired by GSR are classified by Immune Particle Swarm Optimization, obtaining a high average when classifying different emotions from the conductance of the skin. The study presented in [21] shows a method based on Support Vector Regression for recognizing emotions by combining different devices.

Continuing with the differentiation of the emotional state, literature includes other studies like [22], where different devices are combined so that, by means of Cross-Correlation and Fisher, it is possible to distinguish six different kinds of emotions.

In [23], applying a method based on Principal Component Analysis (PCA) to reduce the dimension of the GSR data is proposed, saving as much information as possible. The devices described above are also used in the analysis of different bio signals [24].

## 3. Methods

In order to develop GSR, it is necessary to use a mechanism to send the data via ZigBee as well as the corresponding algorithm to determine the stress level in accordance with the different tests. The different methods used in this first study can be seen below:

### 3.1. Hardware

**Figure 2.**
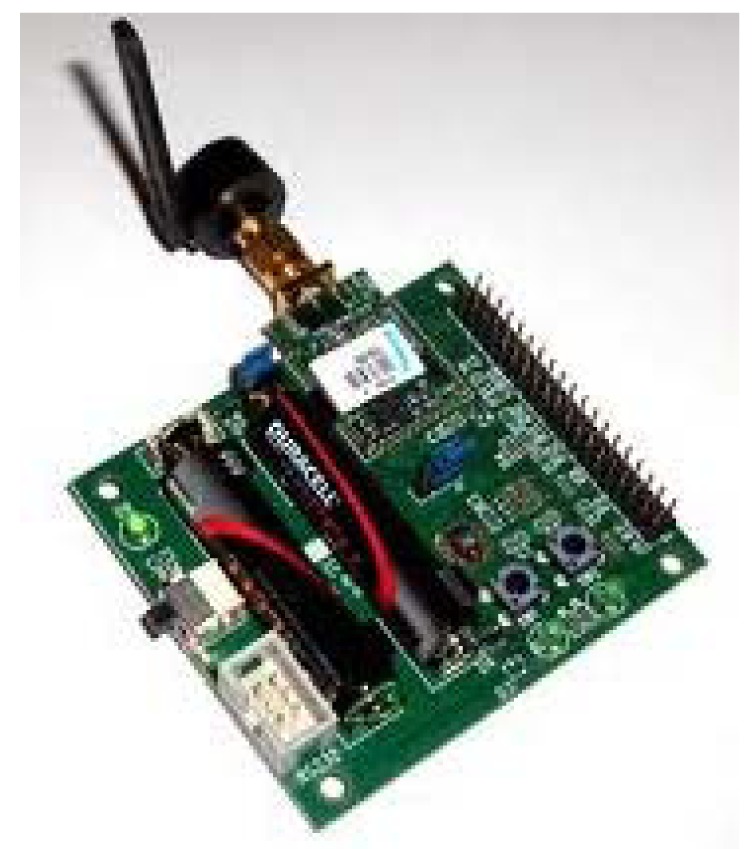
Jennic board

We use Jennic JN-5148 boards (Figure 2) for data acquisition and its subsequent submission to the computer. These were chosen because of their ease of implementing a communication protocol between the coordinator board and the sensor board and because they are part of the ZigBee Alliance. Other devices like those used in [5] were not appropriate for developing a global domotic system. The resolution of the Analogic to Digital Converser is appropriate for the needs of the device.

The output signal of the (*Vo*) device is connected to pin 34 of the sensor board, while the reference signal is connected to number 40. Through the ZigBee communication protocol, the sensor board sends the data to the coordinator board, which, by means of a USB with a JWT terminal, sends the data to a computer. The weight and the size of these boards are quite small, so they can easily be implemented in the same kit as the device.

### 3.2. Signal

After doing some tests, it was seen that the Analogic Digital Converser saturates at 2.35 V. It is an ADC of 12 bits, so the resolution is:

(1) $$\frac{2.35V}{4096}=0.573mV$$

The galvanic skin response oscillates between 10 kΩ and 10 MΩ [25,26], as it can be seen in existing studies about the skin conductance obtained from different applied voltages [27,28]. After initial contact with the subjects, we established an input tension of 1.8 V. We took measurements from our circuit, using different resistances that are within the range of skin resistance (Table 1). These values were chosen in order to know the theoretical behavior of the output voltage, depending on skin conductance. The different values of Rs (Table 1) are determined by the combination of different real resistances.

As the board's ADC has a resolution of 0.573 mV and the minimum tension is 136 mV, an operational amplifier does not have to be included. We can also observe that the differences between some resistance values and others are higher than the ADC resolution. There are GSR devices which use an amplifier before the ADC [6].

### 3.3. Trials

**Figure 3.**
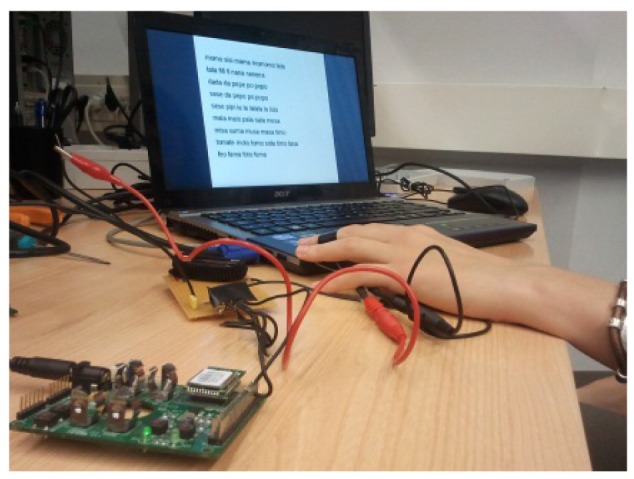
Prototype and test.

Different tests were conducted in order to verify the behavior of the GSR device:

- In a calm state, trying to feel relaxed.
- Trying to be nervous: at the second stage, we asked the subjects to think about something which makes him nervous or produces anxiety.
- Taking in air and expelling it forcefully: the subject is relaxed and, after one minute, he is asked to take in air and expel it, trying to push himself as hard as possible. As the result of this, the nervous system indicates to the sweat glands that an effort has been made.
- Mathematical operations: the subject is relaxed and, after one minute, the computer shows him different mathematical operations. The subject is asked to feed the different results into the computer.
- Reading: after 90 seconds, the screen shows some words that the user has to read as fast as possible (Figure 3).
- Another test where the computer shows different images to the subject was also used. Some of these pictures are “emotional” (they should affect the subject's emotional state) and the others are “neutral” (they have no influence on the subject). It was supposed that the emotional ones would provide a response, but, after trying with some subjects, we have not included this because the results were insignificant.

Table 2 shows the places where the different studies took place. The studies have been made at the subject's home and office because GSR is intended to work in daily situations.

### 3.4. Matlab

In order to analyze and manipulate the different data, we use Matlab.

### 3.5. WEKA

To verify the different tests, we used the WEKA learning machine for testing the following methods: Bayesian Network, J48 and Sequential Minimal Optimization (SMO). They were chosen because there are studies that have obtained good results with them [29]. A Cross-Validation method was used to evaluate the different results.

## 4. System Design

We now present different diagrams showing the application's system design.

### 4.1. High Level Design

**Figure 4.**
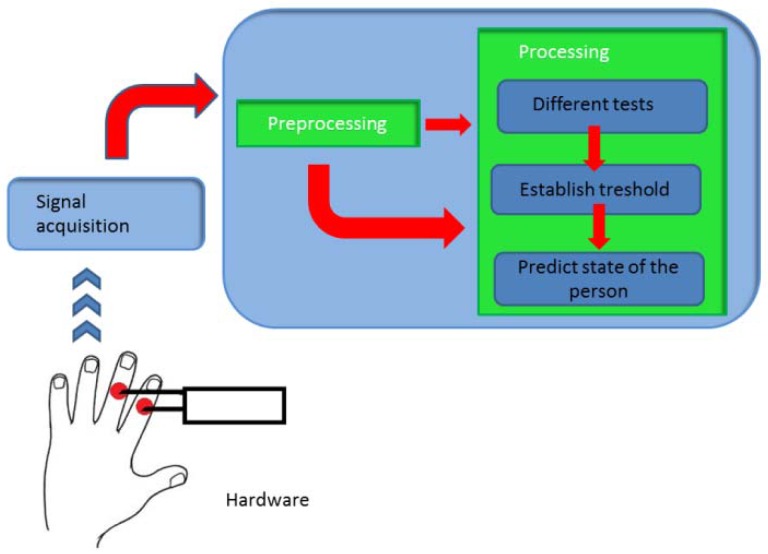
General diagram.

The main application performs the operations on the Figure 4.

**Figure 5.**
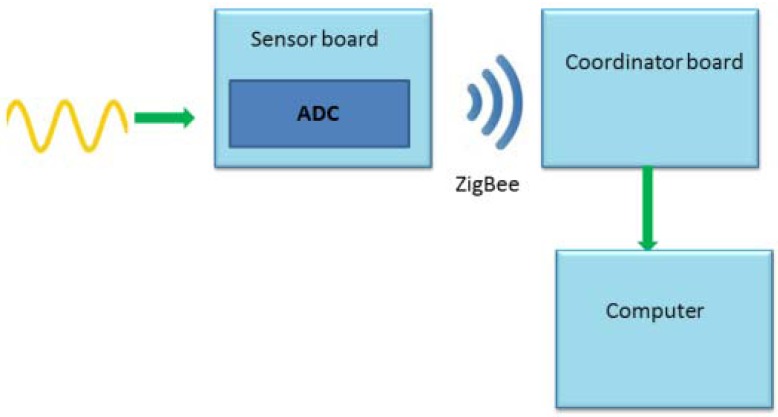
Acquisition diagram.

The data acquisition and subsequent sending of information to the computer is done by two different boards, which are connected to each other via ZigBee (see Figure 5).

**Figure 6.**
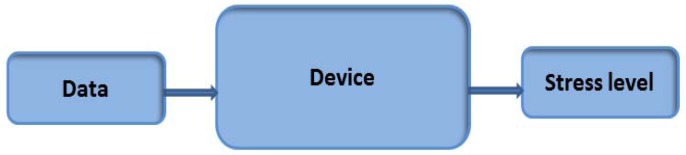
Device function.

The device built performs the operations of the Figure 6:

The GSR electrodes collect the skin resistance and, after this, the device can determine the person's stress level.

### 4.2. Processing Stage

**Figure 7.**
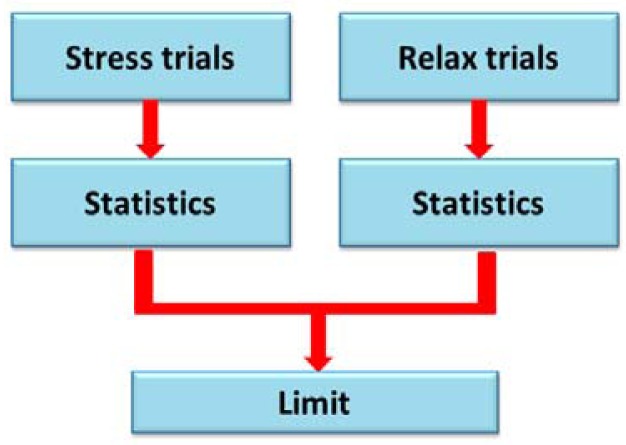
Processing stage.

At this stage (Figure 7) it is necessary to develop an algorithm which must be able to differentiate between the stress levels.

We separate the data according to stress situations and relax situations.

### 4.3. Low Level Design

#### Hardware

**Figure 8.**
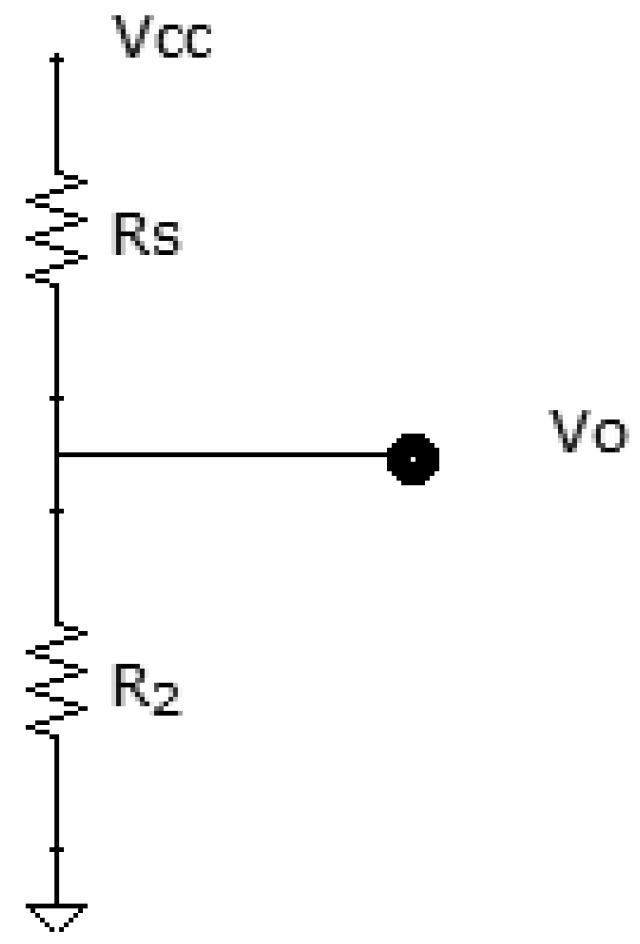
Voltage divider.

A person's skin acts as a resistance to the passage of electrical current. By placing two electrodes on the fingers, we can calculate the Galvanic Skin Response (GSR). To find out this value, we use one resistance, as it can be seen in Figure 8, in series with the skin resistance, to form a voltage divider.

(2) $$Vo=\frac{R2}{Rs+R2}\text{Vcc}$$

where R_s_ is the resistance of the skin.

It can be observed that the *Vo* output tension is inversely proportional to the value of the skin resistance. The more stressed the person is, the more his hands will sweat, so his resistance will decrease. Therefore, we can conclude that the more stress the person is under, the higher output voltage will be.

**Figure 9.**
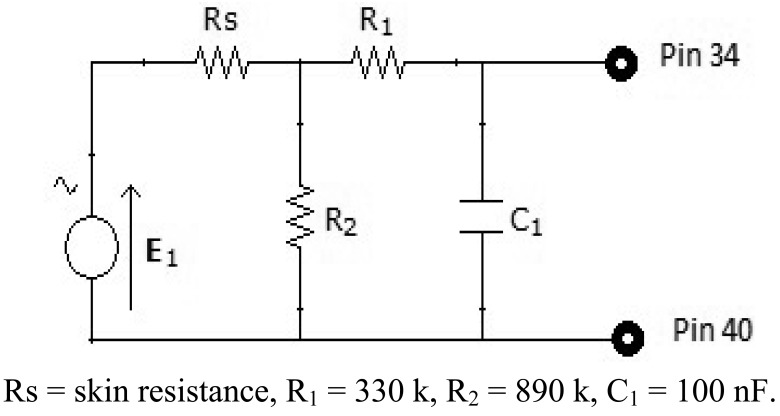
General circuit.

It also includes a low-pass filter made by a capacitor and a resistance to filter the high frequencies. The resulting circuit in Figure 9.

**Figure 10.**
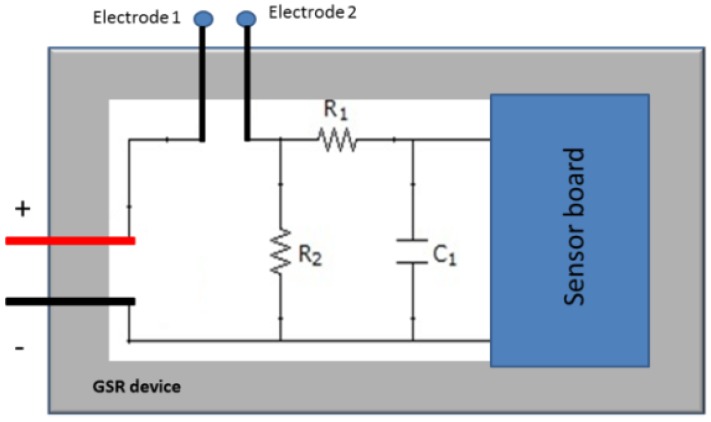
Device.

We will use a 1.5 V battery of as supply. The device, Figure 10, will have the following form:

## 5. Results

We have conducted several tests in order to change the emotional state of the subjects. Knowing the moments when the person should be stressed and the ones where he should not, we can analyze each kind of data separately. We have used 16 subjects aged between 23 and 56 (eight women and eight men). The sample rate is 4 Hz. Once the data have been obtained, we have smoothed them with an average size-5 window.

All the users have done the following tests:

- Staying relaxed
- Mathematical operations
- Breathing deeply
- Reading as fast as possible

Table 3 shows the output voltage averages for the different tests in those cases where the differences are better appreciated.

Following it can be seen the answers to different situations. Figures 11, 16 and 17 show the variation of the output voltage when the users are reading; Figures 12 and 15 represents the effort doing mathematical operations and Figures 13, 14 and 18 are the answer of the user breathing deeply.

**Figure 19.**
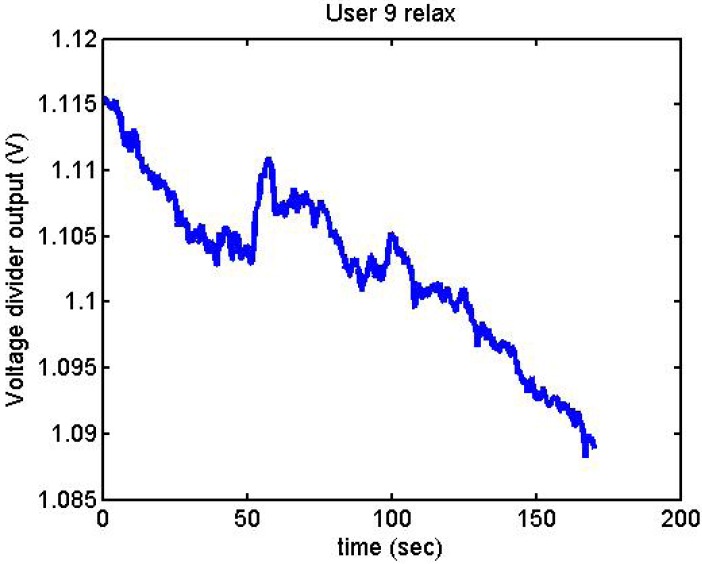
Output voltage of User 9 being relaxed.

**Figure 20.**
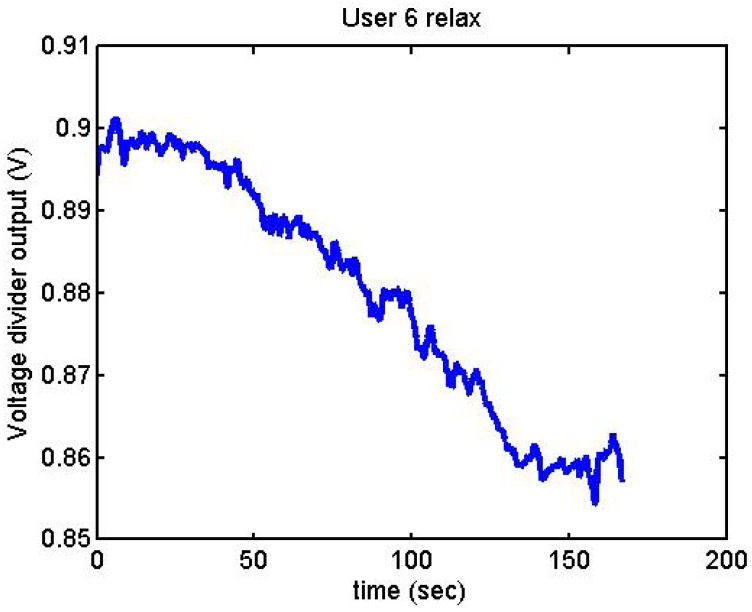
Output voltage of User 6 being relaxed.

**Figure 21.**
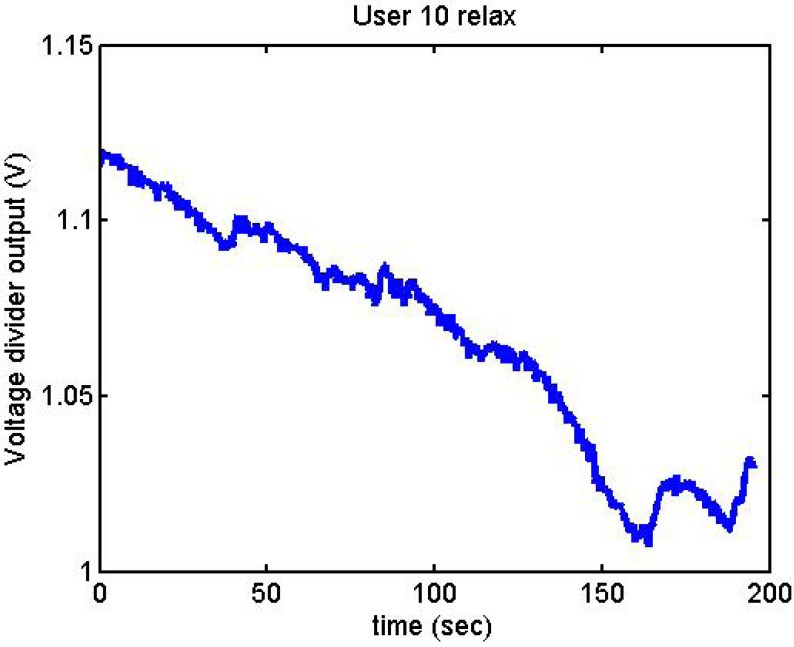
Output voltage of User 10 being relaxed.

**Figure 22.**
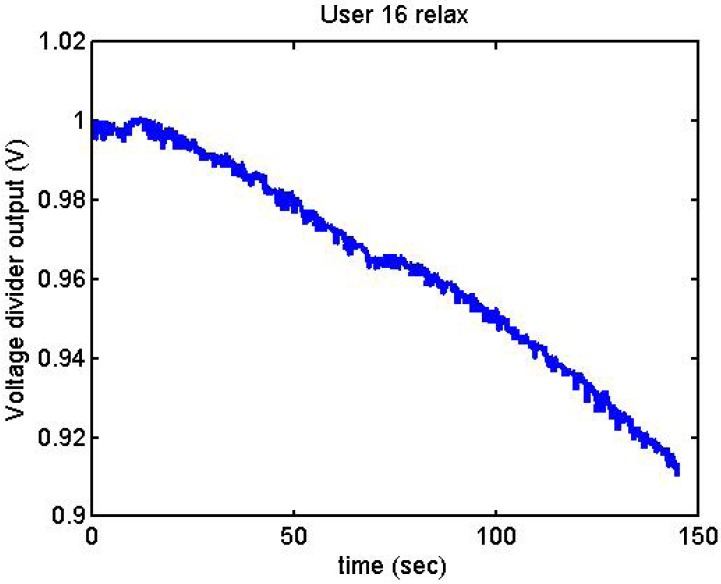
Output voltage of User 16 being relaxed.

It has been appreciated that when the participants were asked to relax, there was a decrease in the output voltage for that period of time. Figures 19–22 show the decreasing of the signal when the user is relaxed.

The Table 4 shows whether changes have been appreciated in these four tests for each user.

**Figure 23.**
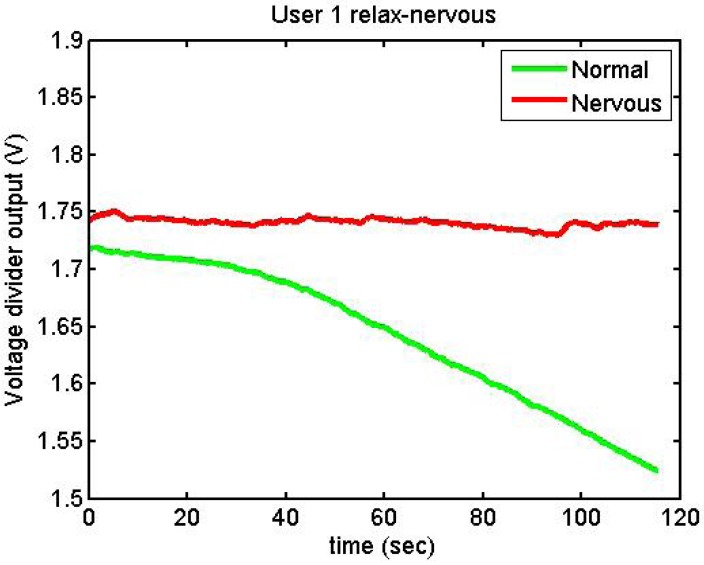
Output voltage of User 1 trying to be relaxed and nervous.

**Figure 24.**
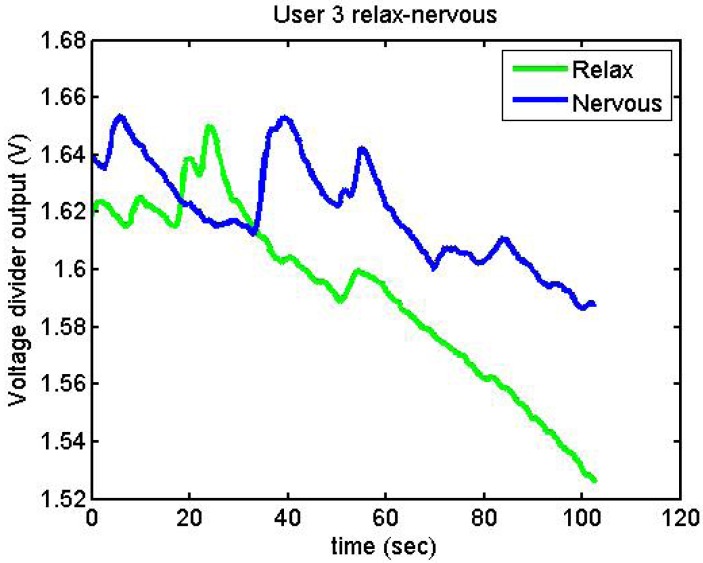
Output voltage of User 3 trying to be relaxed and nervous.

With this data, we obtained an average success rate of 76.56%. The users who had done some trials beforehand (Users 1, 2, 3 and 12), were more successful than the rest. Additionally, there are users who were asked to think of something that makes them nervous for a later comparison (Table 5):

Figures 23 and 24 show the responses of User 1 and User 3, trying to be nervous and relaxed

**Figure 25.**
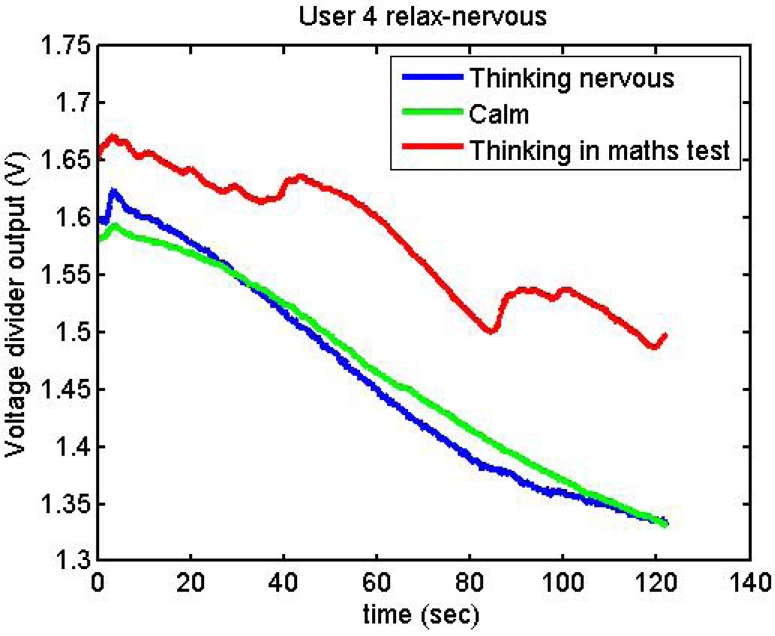
Output voltage of User 4 trying to be relaxed and nervous.

**Figure 26.**
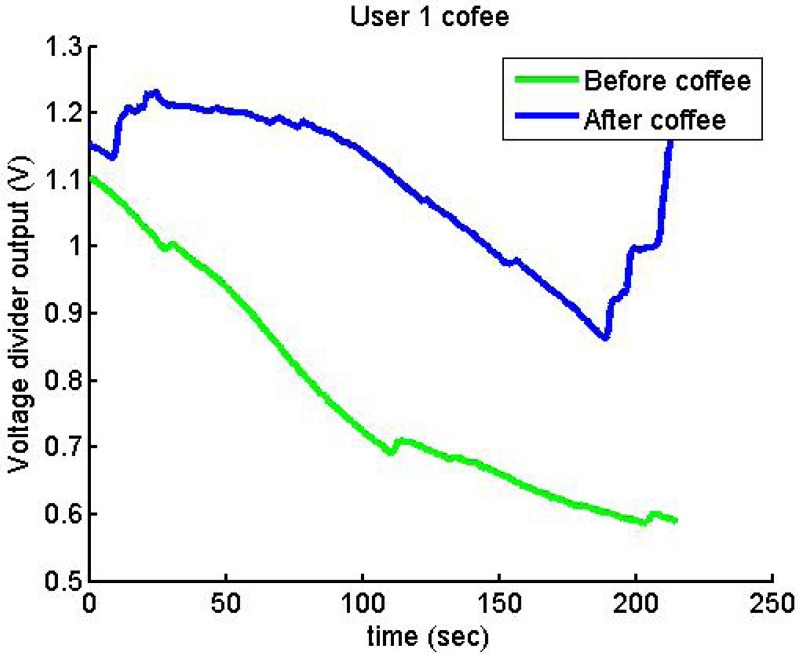
Output voltage of User 4 before and after drinking coffee.

**Figure 27.**
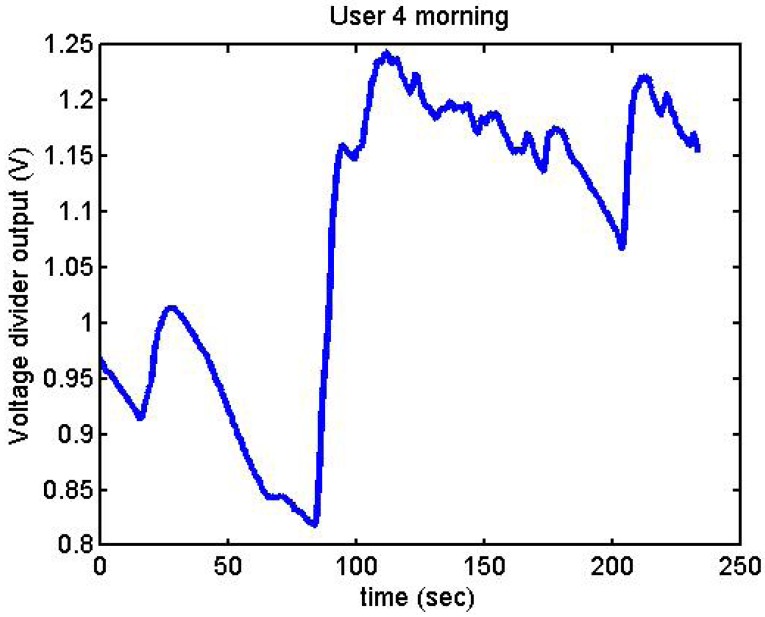
Output voltage of User 4 in the morning.

User 4 was a special case: after doing the stage where she had to think about something that makes her nervous, she had said that she could not do so. We then told her that the next stage was mathematical operations, something which made her nervous (Figure 25). Therefore, we decided repeat the acquisitions:

Figures 26 and 27 show additional situations tested by the GSR:

After drinking coffee, User 1 presents a higher output voltage.

While User 4 was relaxed, she became nervous, which is why an increment in output voltage can be appreciated.

**Figure 28.**
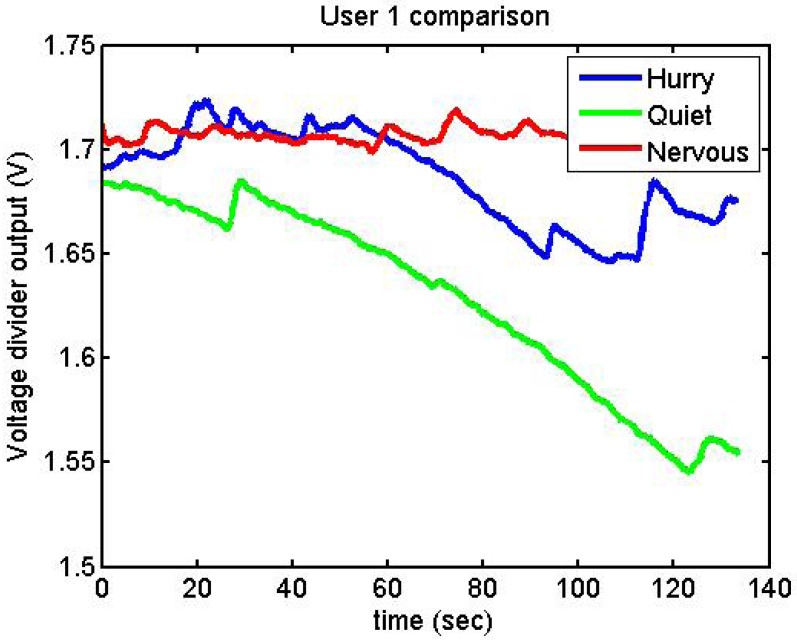
Output voltage of User 1 in different situations.

Figure 28 represents the following three situations:

- Upon arrival at the department, done fairly quickly (blue).
- After a while, much more calmly (green).
- In the afternoon, thinking about something she had to do, and that made her nervous (red).

**Figure 29.**
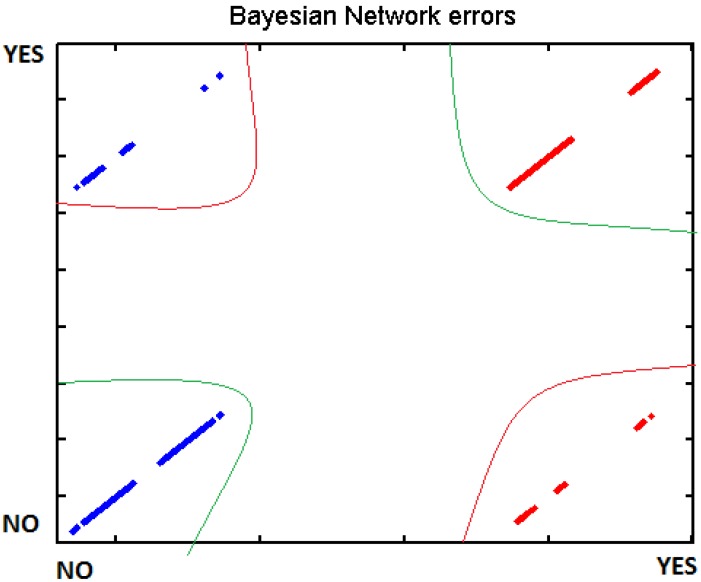
Classifier error BayesNet.

**Figure 30.**
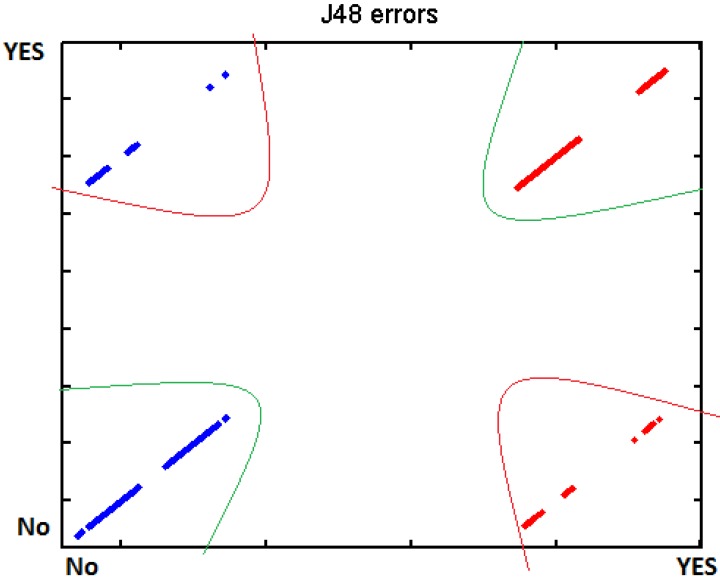
Classifier error J48.

**Figure 31.**
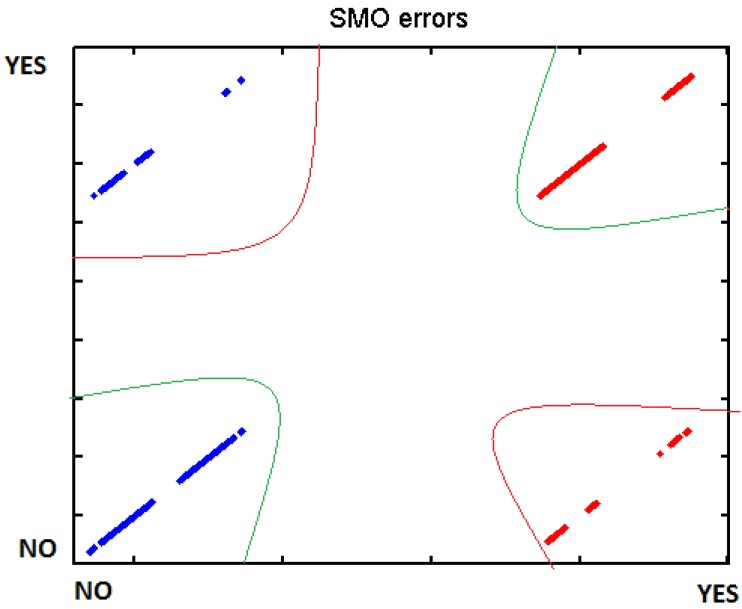
Classifier error SMO.

**Figure 32.**
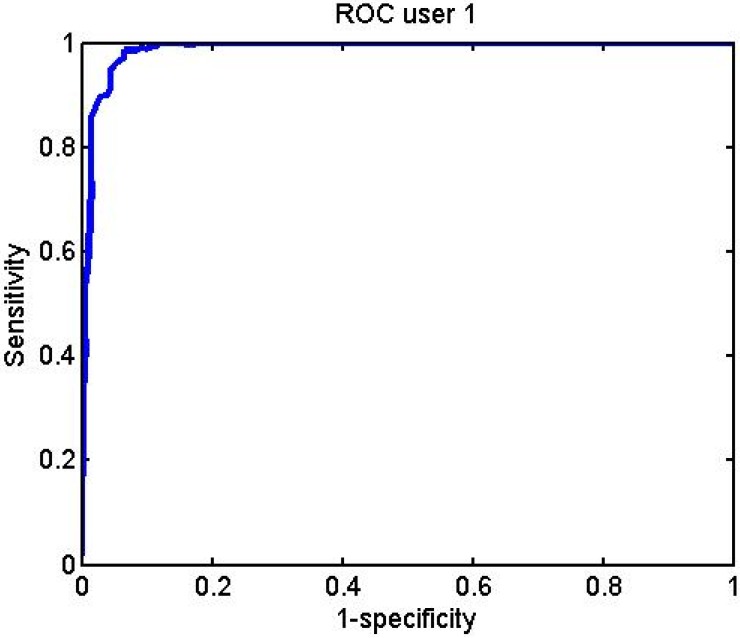
User 1 BayesNet ROC curve.

**Figure 33.**
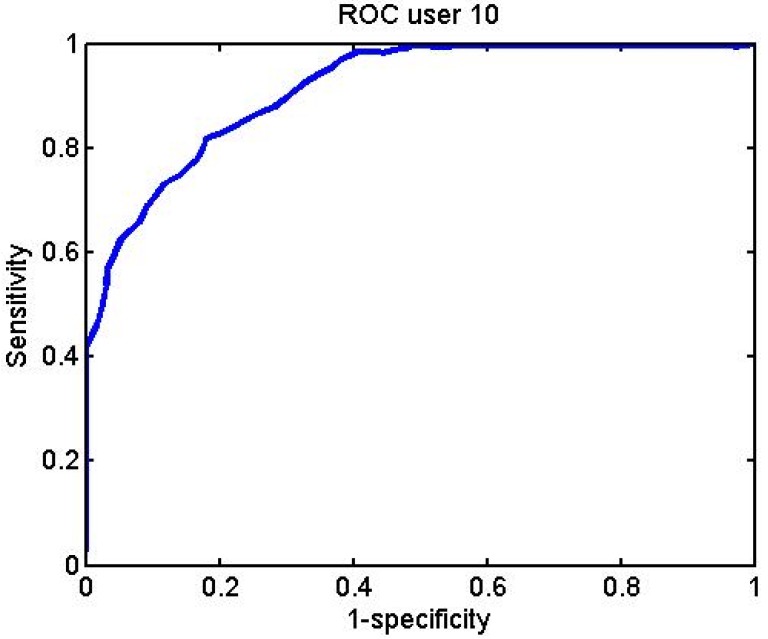
User 10 BayesNet ROC curve.

**Figure 34.**
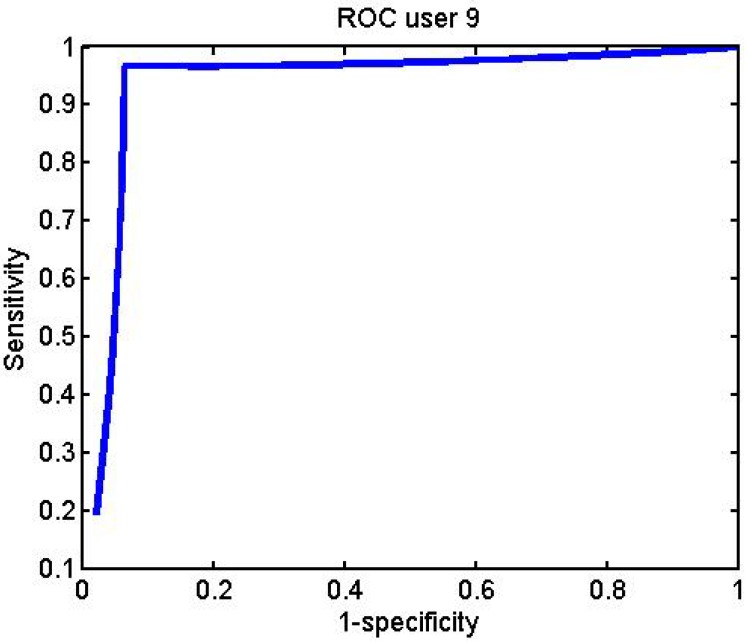
User 9 J48 ROC curve.

**Figure 35.**
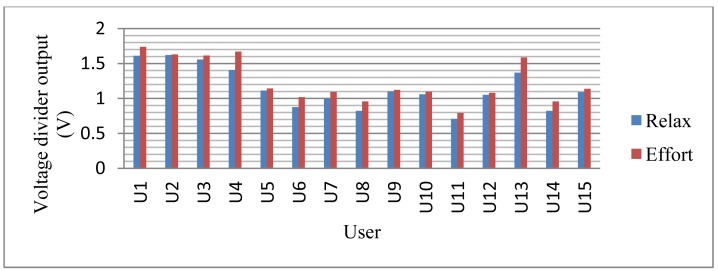
Average comparison between being relaxed and making an effort.

**Figure 36.**
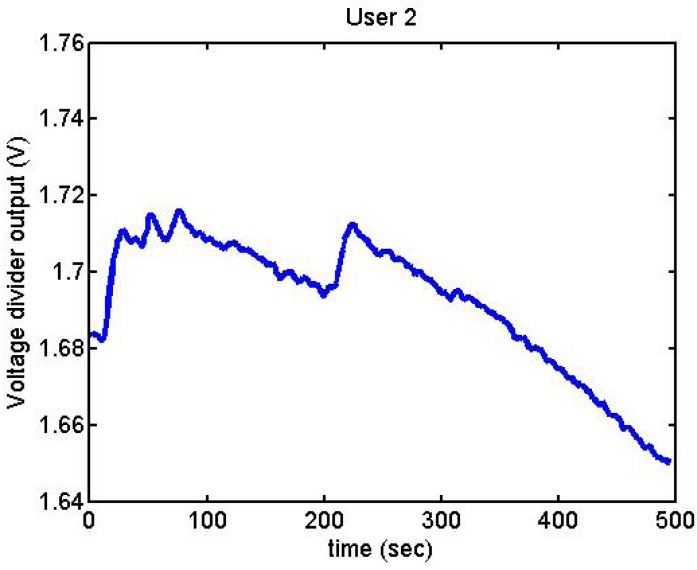
Output voltage of User 2 at the prediction stage.

**Figure 37.**
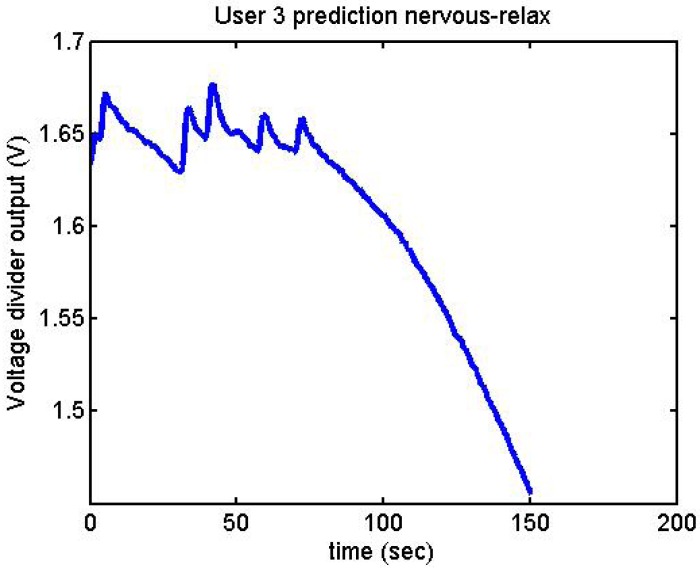
Output voltage of User 3 at the prediction stage, nervous-relaxed.

**Figure 38.**
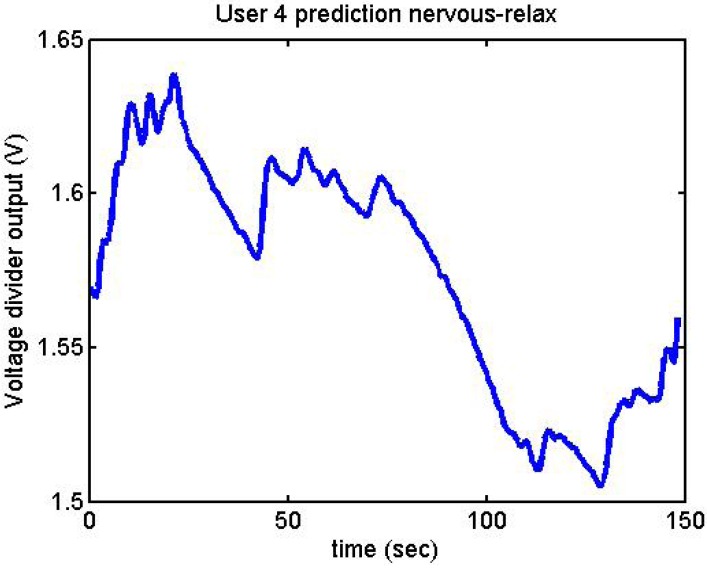
Output voltage of User 4 at the prediction stage, nervous-relaxed.

**Figure 39.**
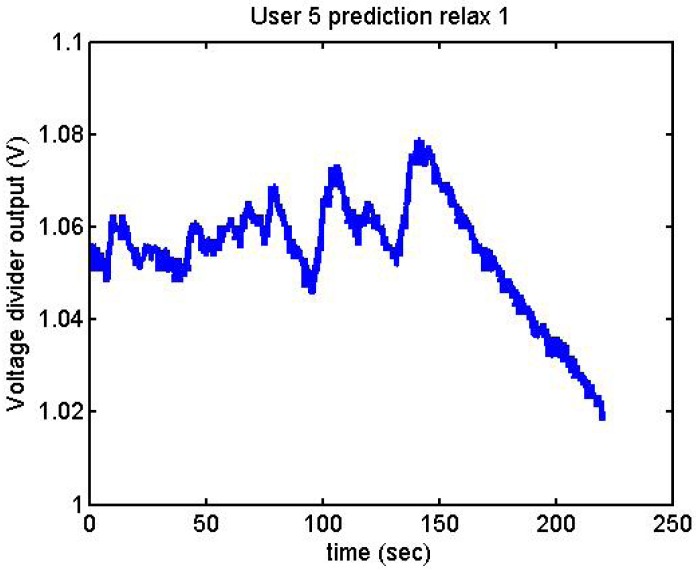
Output voltage of User 5 at the prediction stage, relax 1.

**Figure 40.**
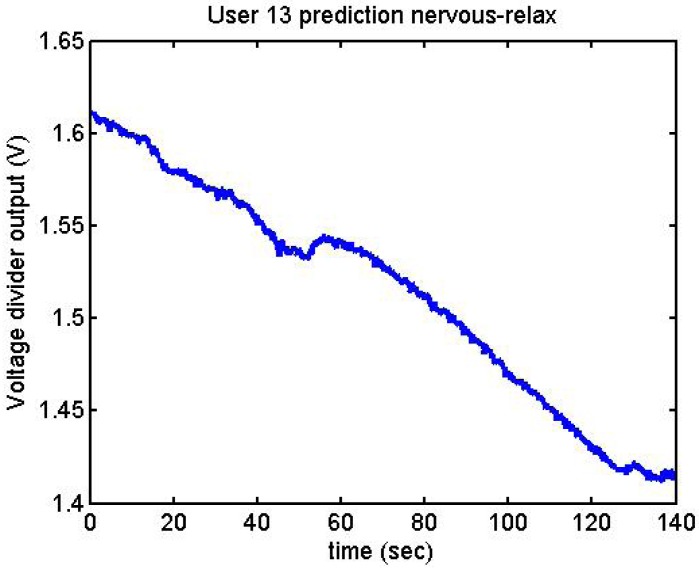
Output voltage of User 13 at the prediction stage, nervous-relaxed.

In order to verify the different tests, we introduced the different data to WEKA machine learning, using BayesNet, J48 and SMO. There separated those measurements which are supposed to represent an effort from those where the user was relaxed. The data have been processed individually for each participant due to the fact that each one has got different thresholds. Cross-Validation was used to test the different classifiers. Below, in Table 6, we present the different results:

Confusion matrix is shown in Table 7:

Figures 29–31 show the graphs of the error classifier:

Figures 32–34 represent the graphs showing the ROC curve for some of the different subjects:

Below is a comparative graph (Figure 35) with the results obtained from the different subjects, showing the averages when relaxed and when in situations requiring effort:

Table 8 contains the different voltages between relaxing situations and effort situations:

We did more trials for Users 2, 3, 4, 5 and 13 in order to determine whether they are stressed or not. In these trials they were asked to feel both relaxed and nervous. After talking to them, they concluded that most of them were not able to make themselves nervous (Figures 36–40). Because of this, the measurements were not treated as they were supposed to be, but as according to how the participant felt. The results are reflected in Tables 9–13.

Table 14 shows the classification average.

We have established the following limit to differentiate being relaxed from being nervous for an initial study:

(3) $$\text{Limit}=\frac{\text{stress average}\times 0.6+\text{relax average}\times 0.4}{2}$$

**Figure 41.**
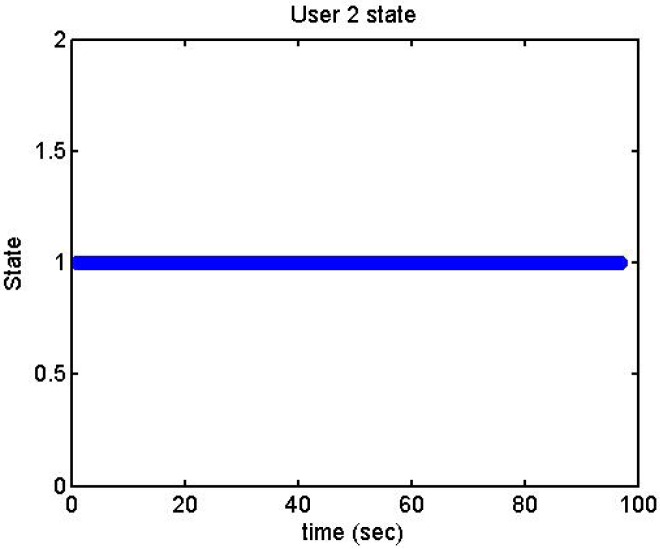
State of the User 2 at the prediction stage.

**Figure 42.**
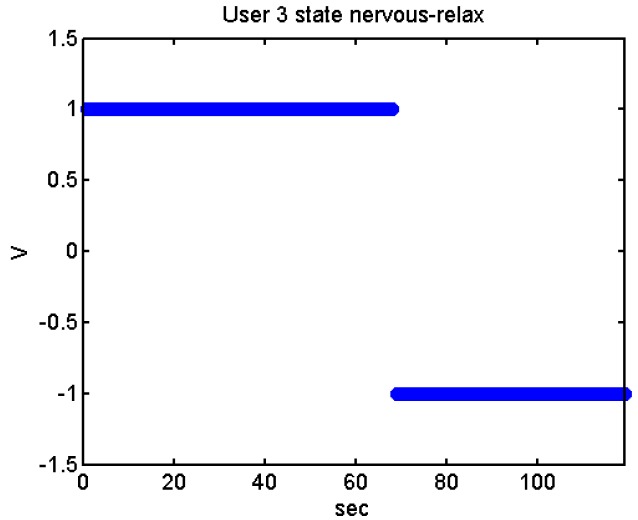
State of the User 3 at the prediction stage, nervous-relaxed.

**Figure 43.**
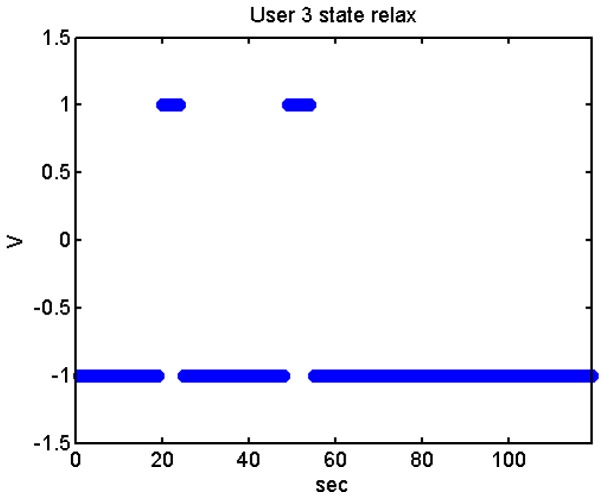
State of the User 3 at the prediction stage, relaxed.

**Figure 44.**
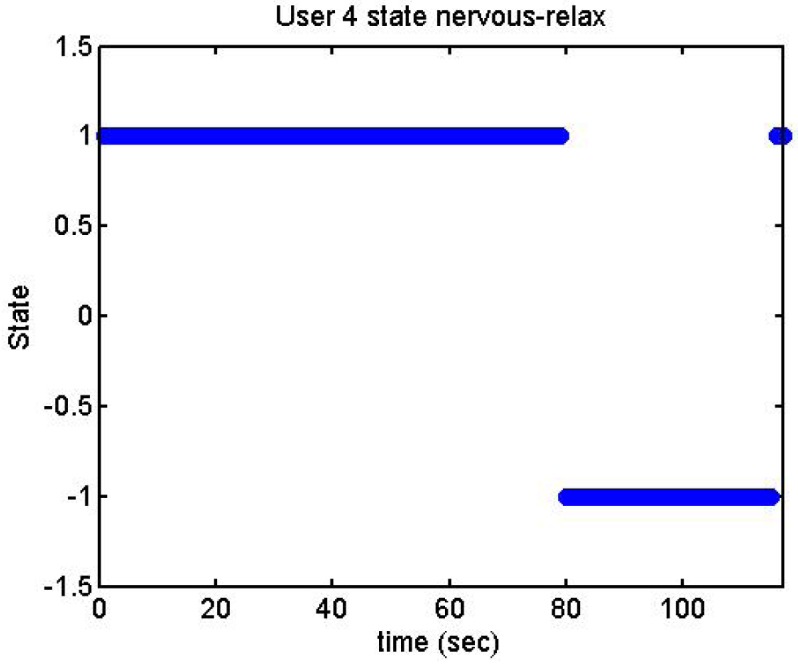
State of the User 4 at the prediction stage, nervous-relaxed.

**Figure 45.**
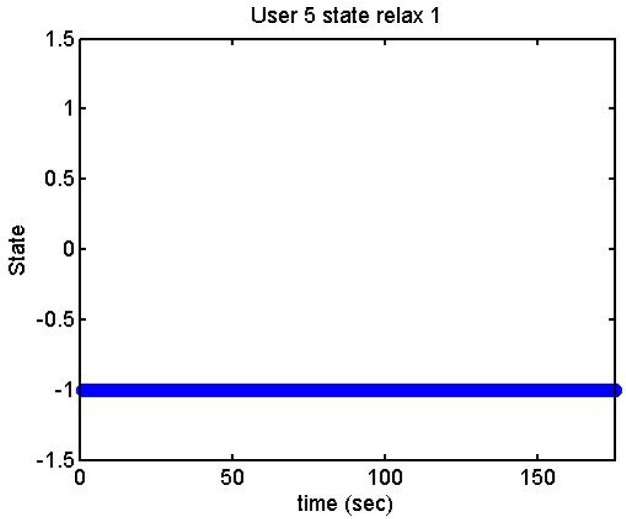
State of the User 5 at the prediction stage, relaxed 1.

**Figure 46.**
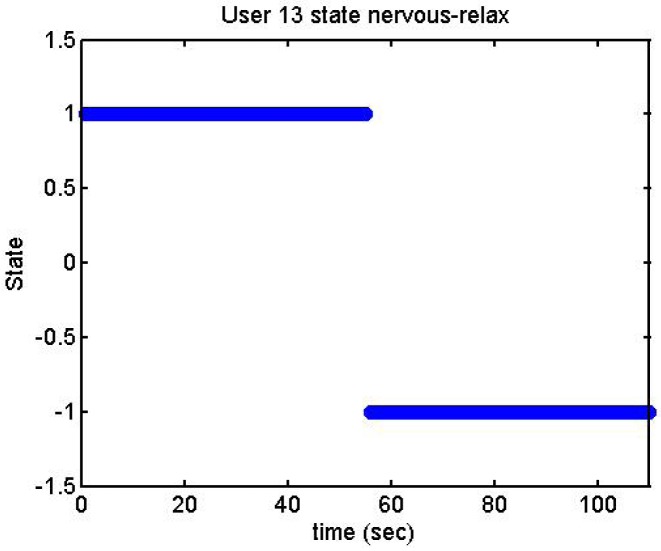
State of the User 13 at the prediction stage, nervous-relaxed.

We have separated the data in windows of 10 seconds, overlapping 5 seconds. If the average is higher than the limit, the result is 1, if is lower, the result is −1 (Figures 41–46). These are the results:

## 6. Discussion

The main part of this study involved the design of a device which is able to detect skin resistance in different situations. It also includes an initial threshold between being stressed and being relaxed, but it is not the algorithm that is going to be implemented in the final application.

With the different graphs, it can be observed that signals increase or decrease depending on the effort or the mental situation of the user. User 4, User 2, and User 3 had done some previous tests before these results. This may be the explanation for why they present more clarity in their graphs.

The main problem is that, for the moment, we cannot differentiate being stressed from making an effort. This is clearly seen in User 13's last graph, where a laugh presents a similar response to feeling stressed. Apart from the reflected trials, we also collected data while the user was playing different games, such as Tetris or PacMan. However, significant results were not obtained, so they are not included in this study. There are other studies [30] which have used different games in longer tests, and they have obtained good results.

## 7. Conclusions

The GSR device detects whether there has been an effort or a different situation from being relaxed with a success rate of 90.97%. It has been observed that participants who had done some trials before obtained the highest difference; so the average could be higher if the user is familiarized with the device. The next stage is to design an algorithm in order to establish the threshold between different emotional situations because this first algorithm does not distinguish between being stressed or making an effort. Two tasks lying ahead of us are:

- Improving the algorithm to establish more reliable thresholds;
- Using different tests for the calibration state: conducting tests that last longer [17,30].

## Figures and Tables

**Figure 11.**
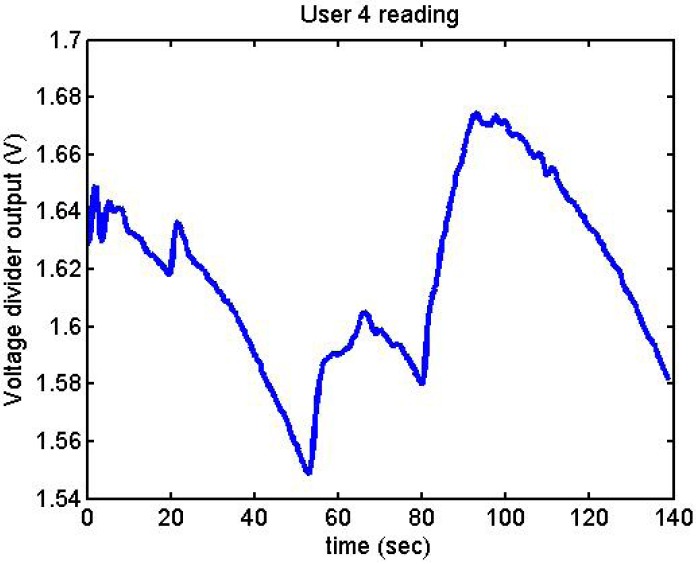
Output voltage of User 4 reading.

**Figure 12.**
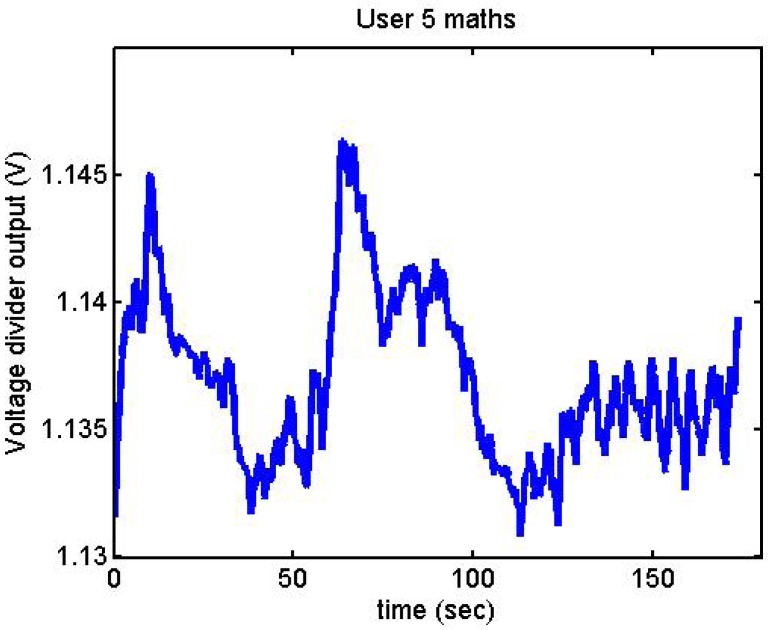
Output voltage of User 5 doing mathematical operations.

**Figure 13.**
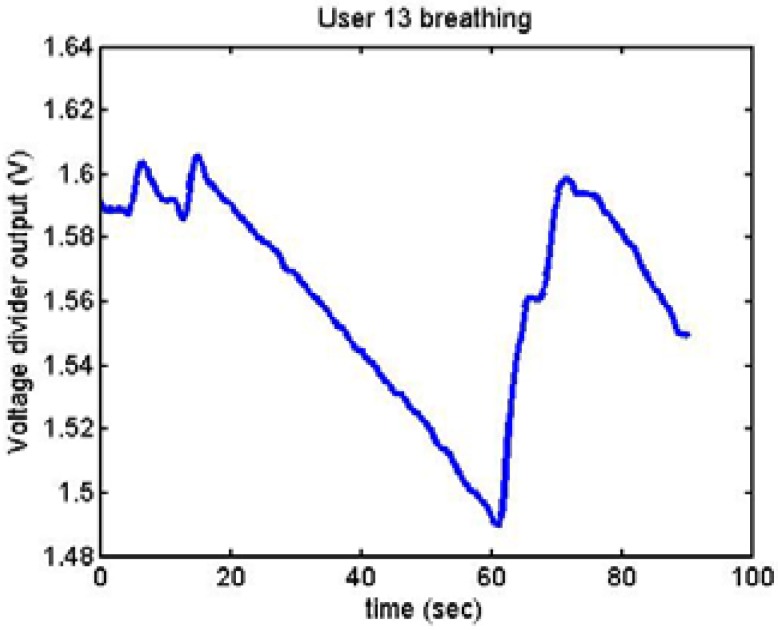
Output voltage of User 13 breathing.

**Figure 14.**
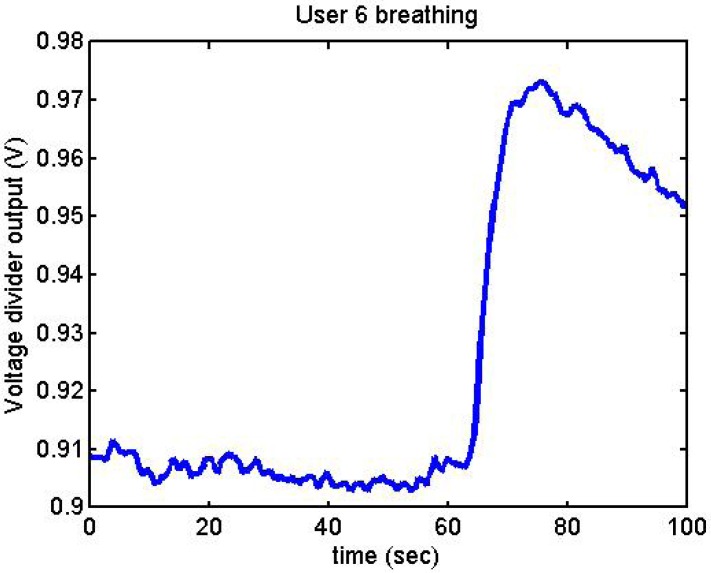
Output voltage of User 6 breathing.

**Figure 15.**
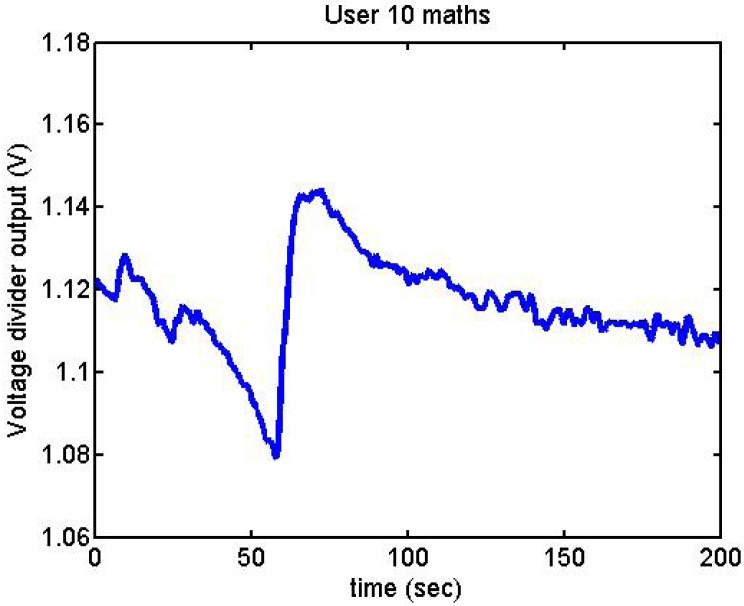
Output voltage of User 10 doing mathematical operations.

**Figure 16.**
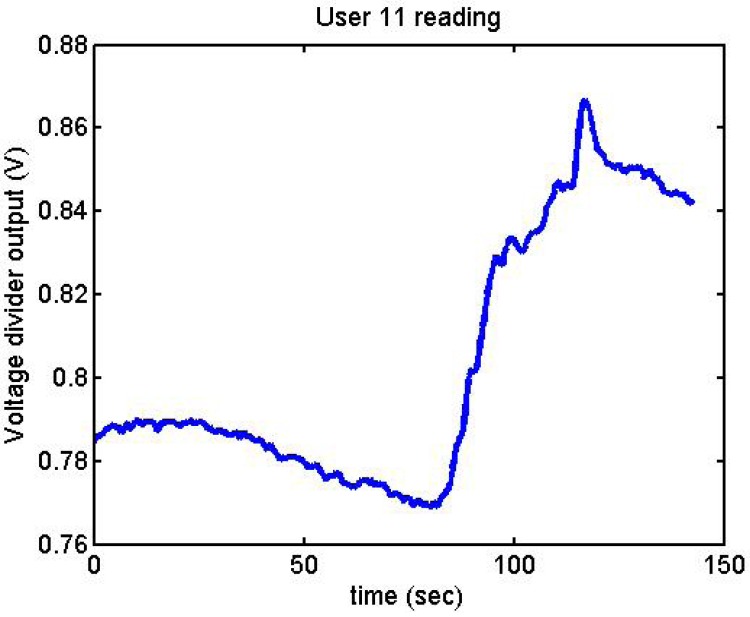
Output voltage of User 11 reading.

**Figure 17.**
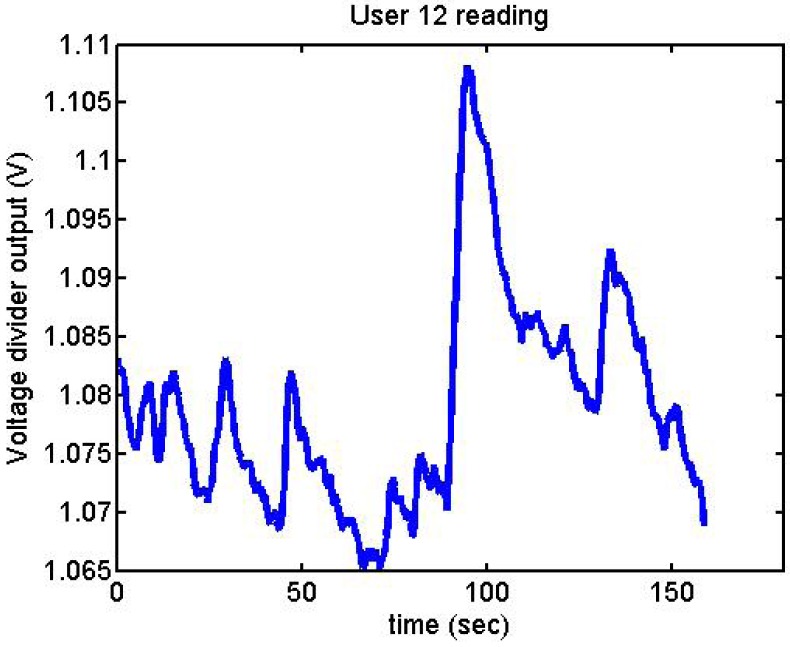
Output voltage of User 12 reading.

**Figure 18.**
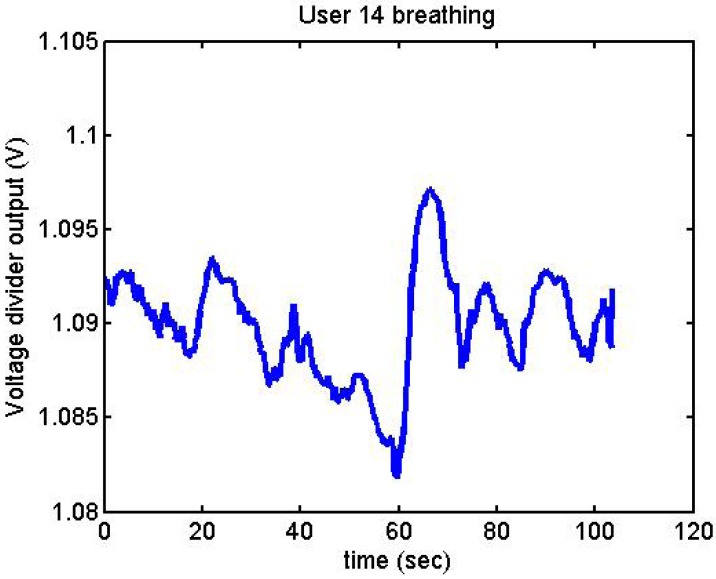
Output voltage of User 14 breathing.

**Table 1.** Different values of the resistances.

**Rs**	**Vout**

10 k	1.755 V
49.5 k	1.677 V
100 k	1.587 V
200 k	1.434 V
560 k	1.054 V
760 k	0.923 V
1 M	0.813 V
3.3 M	0.357 V
9.93 M	0.136 V

**Table 2.** Places of the tests.

**User 1**	Work	**User 9**	Home
**User 2**	Work	**User 10**	Home
**User 3**	Work	**User 11**	Home
**User 4**	Work	**User 12**	Work
**User 5**	Home	**User 13**	Home
**User 6**	Home	**User 14**	Work
**User 7**	Home	**User 15**	Work
**User 8**	Home	**User 16**	Home

**Table 3.** Average of different situations.

	**Relax(V)**	**Operations (V)**	**Breathing (V)**	**Reading (V)**
	
**User 4 (25)**	1.4068	1.6945	1.6476	1.6712
**User 5 (30)**	1.1123	1.1383	1.1484	1.1426
**User 6 (27)**	0.876	1.0381	0.9567	1.0609
**User 7 (24)**	1.0011	1.0868	1.0786	1.1176
**User 8 (26)**	0.8238	0.9904	0.8864	0.9975
**User 9 (26)**	1.101	1.1145	1.1105	1.1439
**User 10 (27)**	1.060	1.1197	1.0695	1.1022
**User 11 (55)**	0.7096	0.7546	0.7840	0.8408
**User 12 (28)**	1.0529	1.0685	1.0893	1.0856
**User 13 (23)**	1.3699	1.5542	1.5599	1.6468
**User 14 (30)**	0.8238	0.9904	0.8864	0.9975
	

**Table 4.** Success of each test by subject.

	**Reading**	**Breathing**	**Operations**	**Relaxing**
	
**User 1**	YES	YES	YES	YES
**User 2**	NO	YES	YES	YES
**User 3**	YES	YES	YES	YES
**User 4**	YES	YES	YES	YES
**User 5**	NO	YES	YES	YES
**User 6**	YES	YES	YES	YES
**User 7**	NO	YES	NO	YES
**User 8**	YES	NO	NO	YES
**User 9**	YES	NO	YES	YES
**User 10**	NO	YES	YES	YES
**User 11**	YES	YES	NO	YES
**User 12**	YES	YES	YES	NO
**User 13**	NO	YES	NO	YES
**User 14**	YES	NO	YES	YES
**User 15**	NO	NO	YES	YES
**User 16**	YES	YES	YES	YES
	
**Total success**	**10**	**12**	**12**	**15**
% **success**	**62.5**	**75**	**75**	**93.75**
	

**Table 5.** Differences between trying to be relaxed and trying to be nervous.

	**Relaxed(V)**	**Nervous(V)**
	
**User 1 (26)**	1.6118	1.7396
**User 2 (26)**	1.5535	1.5379
**User 3 (24)**	1.5576	1.6153
**User 4 (25)**	1.4068	1.3839
**User 5 (30)**	1.1123	1.1266
**User 15 (26)**	1.0902	1.1388
	

**Table 6.** Global results.

	**BayesNet**	**J48**	**SMO**
	
**Relative absolute error**	15.86%	16.91%	17.17%
**Root relative squared error**	26.77%	27.20%	41.61%
**Correctly classified**	93.73%	93.79%	91.95%
**Incorrectly classified**	6.27%	6.21%	8.05%
	

**Table 7.** Confusion matrix.

**BayesNet**			**J48**			**SMO**		
	P	N		P	N		P	N
T	**8861**	**1014**	T	**8867**	**1008**	T	**8546**	**1329**
F	**442**	**10347**	F	**435**	**10354**	F	**497**	**10292**

**Table 8.** Difference between being relaxed and effort situations.

	**Calm (V)**	**Effort (V)**	**Diference (%)**
	
**User 1**	1.6118	1.7396	7.929
**User 2**	1.6216	1.6309	0.5735
**User 3**	1.5576	1.6153	3.70445
**User 4**	1.4068	1.6711	18.7873
**User 5**	1.1123	1.1431	2.7690
**User 6**	0.876	1.0186	16.2747
**User 7**	1.0011	1.0943	9.3131
**User 8**	0.8238	0.9581	16.3025
**User 9**	1.101	1.123	1.9952
**User 10**	1.060	1.0971	3.5129
**User 11**	0.7096	0.7931	11.7718
**User 12**	1.0529	1.0811	2.6814
**User 13**	1.3699	1.587	15.84548
**User 14**	0.8238	0.9581	16.3023
**User 15**	1.0902	1.1388	4.4499
	

**Table 9.** Results of the different methods for User 2.

**User 2**			
	**Trial 1: stressed**		
	**BayesNet**	**J48**	**SMO**
	
**Relative absolute error**	0.95%	0.00%	0.00%
**Correctly classified**	100.00%	100.00%	100.00%
**Incorrectly classified**	0.00%	0.00%	0.00%
	

**Table 10.** Results of the different methods for User 3.

**User 3**			
	**Trial 1: stressed and relaxed**		
	**BayesNet**	**J48**	**SMO**
	
**Relative absolute error**	30.29%	43.61%	21.35%
**Correctly classified**	85.55%	85.55%	89.37%
**Incorrectly classified**	14.45%	14.45%	10.63%
	
	**Trial 2: relaxed**		
	**BayesNet**	**J48**	**SMO**
	
**Relative absolute error**	38.05%	42.40%	29.87%
**Correctly classified**	84.00%	84.00%	90.90%
**Incorrectly classified**	16.00%	16.00%	9.10%
	

**Table 11.** Results of the different methods for User 4.

**User 4**			
	**Trial 1: stressed and relaxed**		
	**BayesNet**	**J48**	**SMO**
	
**Relative absolute error**	29.43%	40.73%	24.91%
**Correctly classified**	85.35%	79.63%	87.54%
**Incorrectly classified**	14.65%	20.37%	12.46%
	
	**Trial 2: relaxed**		
	**BayesNet**	**J48**	**SMO**
	
**Relative absolute error**	0.21%	7.84%	0.00%
**Correctly classified**	100.00%	96.17%	100.00%
**Incorrectly classified**	0.00%	3.83%	0.00%
	

**Table 12.** Results of the different methods for User 5.

**User 5**			
	**Trial 1: relaxed**		
	**BayesNet**	**J48**	**SMO**
	
**Relative absolute error**	0.00%	0.00%	0.00%
**Correctly classified**	100.00%	100.00%	100.00%
**Incorrectly classified**	0.00%	0.00%	0.00%
	
	**Trial 2: relaxed**		
	**BayesNet**	**J48**	**SMO**
	
**Relative absolute error**	0.12%	0.00%	0.00%
**Correctly classified**	100.00%	100.00%	100.00%
**Incorrectly classified**	0.00%	0.00%	0.00%
	

**Table 13.** Results of the different methods for User 13.

**User 13**			
	**Trial 1: stressed and relaxed**		
	**BayesNet**	**J48**	**SMO**
	
**Relative absolute error**	32.50%	35.11%	40.94%
**Correctly classified**	86.96%	86.96%	79.46%
**Incorrectly classified**	13.04%	13.04%	20.54%
	
	**Trial 2: relaxed**		
	**BayesNet**	**J48**	**SMO**
	
**Relative absolute error**	42.17%	45.61%	51.85%
**Correctly classified**	71.43%	71.43%	71.43%
**Incorrectly classified**	28.57%	28.57%	28.57%
	

**Table 14.** Classification average of the different methods.

**BayesNet**	90.37%
**J48**	89.30%
**SMO**	90.97%
